# Editorial: Gender differences and disparities in socialization contexts: How do they matter for healthy relationships, wellbeing, and achievement-related outcomes?

**DOI:** 10.3389/fpsyg.2022.1103425

**Published:** 2022-12-02

**Authors:** Caterina Fiorilli, Daniela Barni, Joyce Endendijk, Jan Retelsdorf

**Affiliations:** ^1^Department of Human Sciences, Libera Università Maria SS. Assunta (LUMSA), Rome, Italy; ^2^Department of Human and Social Sciences, Università degli Studi di Bergamo, Bergamo, Italy; ^3^Child and Adolescent Studies, Utrecht University, Utrecht, Netherlands; ^4^Faculty of Education, Educational Psychology, University of Hamburg, Hamburg, Germany

**Keywords:** gender differences, gender disparities, school, teachers, family, parents, youth, wellbeing

Gender differences and disparities in youth's development, education, and socialization are part of long-standing scientific, political, and public debates. According to the European Institute for Gender Equality (https://eige.europa.eu/), gender disparities refer to differences in women's and men's access to resources, status, and wellbeing, which usually favor men and not rarely are institutionalized through law, justice, and social norms. Despite remarkable advances in furthering the status of women, gender disparities still remain a worldwide challenge, as no country has achieved full gender parity yet (World Economic Forum, 2022). At the current rate of progress, it will take 132 years to close the global gender gap. Gender disparities largely persist in several life domains such as school (e.g., in academic pathway and achievement), work (e.g., career development and wages), and family (e.g., household division and parental expectations of children), and can result in context- and gender-specific problems and maladjustment. It is thus essential to better understand the psychosocial mechanisms underlying gender differences in socialization contexts in order to reduce the risk of harmful disparities and strengthen the factors fostering equitable development opportunities for girls and boys.

With a multiperspective approach, the current Research Topic (RT) aims to contribute to the international debate by offering scientific data and educational and social suggestions for building a social context supporting optimal development of youth, regardless of their gender. The following sections describe the RT's contributions in two sub-themes.

## Gender disparities: From school to university

Most current RT papers allow us to observe how the gender gap in the school context persists in many countries (Austria, Australia, Colombia, Denmark, Germany, Italy, Japan, Quebec, Nigeria, Switzerland, and United States) and at different school-ages. Together these studies highlight the need for extra attention to gender differences in the school context by education staff and policymakers.

A large body of literature is devoted to girls' and boys' attitudes and performance in science, technology, engineering, and mathematics (STEM). In line with Eccles and Wigfield's (2020) situated expectancy-value theory, many sociocultural, contextual, biological, behavioral, and psychological variables may contribute to the widespread under-representation of girls and women in the scientific field and a lower academic self-concept than boys. In this regard, Valls's research has confirmed gender differences in academic self-concept with girls feeling more confident in language learning and boys feeling more confident in mathematics. Furthermore, Valls's research demonstrated that negative social comparison processes could best explain these gender differences, which, in turn, may negatively impact boys' and girls' motivation toward certain academic challenges. Similarly, Andersen and Smith found that the social contexts in schools (i.e., teacher gender stereotypes, comparisons with math achievement of female peers) generates gender differences in young people's self-concept and achievements in math and language. In Hübner et al.'s study clear disparities favoring boys were found for upper secondary school achievements in math and physics and to a lesser extent in biology. These disparities did not increase (nor decrease) after a recent school time reform in Germany that reduced overall school time, which was compensated by increased average instructional time per week. Although, girls' level of stress and wellbeing was negatively affected by this instructional time reform to a greater extent than for boys, which may on a longer term exacerbate existing gender disparities in the school context.

Interestingly, as Froehlich et al. outlined, although there are no gender differences in math ability in young STEM students, expected backlash (i.e., less positive reactions to university major) affected female STEM students' emotions and STEM motivation to a larger extent than male STEM students. Despite the relatively higher level of female students' mathematics achievement than boys, they maintain a weaker math self-concept, negatively affecting the cognitive resources necessary to perform STEM tasks better (Bertrams et al.).

Similarly, Musso et al. focused on STEM-gender stereotypes and assumed that gender disparities become more complex and pronounced when socioeconomic status (SES) is considered. The authors shed light on the unneglectable consideration that higher SES is associated with lower STEM-gender stereotypes. With a different approach to SES, Kuzyk et al. confirmed the interrelationships between SES, nationality, and gender, which may interactively impact students' cognitive performance and self-perceptions of this performance. Additionally, despite evidence that IQ levels are equally distributed between genders, there is a significant gender gap in self-estimated intelligence, with males providing systematically higher estimates than females (Reilly et al.).

How gender-stereotypes and disparities threaten adolescents' mental health and wellbeing is a second Research Topic concerning gender disparities at school. According to Rubach et al., it is not a surprise that male and female students report distinct stressors and mental health troubles contextually observed during the COVID-19 pandemic. Nevertheless, teachers' instructional quality may reduce mental health menaces and enhance students' academic satisfaction. Similarly, Korlat et al. focused on gender role self-concept (i.e., masculine, feminine, androgynous, and undifferentiated) in relation to school-related wellbeing. Their findings showed that an androgynous self-concept might be optimal for academic wellbeing. Furthermore, their study opens urgent reflections on how school staff might approach gender-typed attributes in students.

With a different perspective on the educational setting, the third theme of the RT focuses on the relationships between teachers' gender and their mental health. Kreuzfeld and Seibt shed an interesting light on how male and female teachers differ in terms of working conditions and coping with high work demands, as well as individual factors that promote early retirement. By collecting several types of data from a gender-balanced group of teachers, the authors found that female teachers have a greater tendency to overcommit themselves and have a worse capacity to recover from troubles than male teachers. A second study by Dersch et al. addressed educators' stereotypes regarding STEM and outlined that teachers' misconceptions may impact their students' self-concepts. Preservice teachers' training should thus promote their awareness of gender misconceptions.

The focus on teacher-student relationships was also analyzed in the research by Beißert et al. concerning teachers' reactions to social exclusion among students by considering their gender. Interestingly, teachers were less likely to intervene if a boy was excluded than if a girl was excluded. This study drew attention to male-specific school disparities by showing that also boys can be at risk of being encapsulated in their gender role, which, in turn, may negatively affect their school-adjustment.

Finally, Bluteau et al. analyzed the relationship between students' seating in the classroom and their school-related wellbeing. Flexible classroom seating positively affects girls' wellbeing, while male students take advantage of fixed classroom seating. Thus, seating arrangements, and individual differences in the need for personal space, could contribute the gender gap in wellbeing at school.

An important future direction for research on gender disparities in the school context is to not primarily focus on gender in STEM, but also examine processes related to the underrepresentation of boys and men in HEED (health care, elementary education, domestic sphere; Croft et al., 2015) as well as gender differences in the performance on other school subjects (e.g., language, arts).

## Reducing gender disparities: Start early, at home

For the greater part of childhood and early adolescence, the family is another primary context in which children and youths are socialized about gender and gender roles (e.g., Lawson et al., 2015). Parents engage in numerous *cultural socialization* processes and practices, which expose children to information that helps them to learn about their history, heritage (values, religion, traditions, customs, etc.), and social norms (e.g., what is socially expected from a girl or a boy). One such cultural process among families is parent–child transmission of norms, beliefs, and values which many scholars consider the hallmark of successful intergenerational socialization (Knafo-Noam et al., 2020). Parents widely use perceived social norms and stereotypical beliefs as a reference when socializing children (Tam et al., 2012). This clearly emerged from Barni et al.'s study, which showed a significant relationship between parents' hostile and benevolent sexism and their socialization values (i.e., the values parents want to transmit to their children). The more parents, especially fathers, hold sexist beliefs against women, the more they would like their young adult children to be conservative.

Parents' beliefs translate into daily practices and influence children's development of competencies and motivations. In this regard, Mues et al., involving preschool children, showed that parents' mathematical gender stereotypes (in favor of boys), self-efficacy, and their beliefs on the importance of mathematical activities at home are related to parents' numeracy activities and children's numeracy competencies. The findings supported the assumption of a direct association between children's numeracy competencies and parents' numeracy-related activities for fathers only, but not for mothers. In general, parents' gender-differentiated encouragement of science or language predicts children's later motivations (Shirefley and Leaper) and even career decisions (Endendijk and Portengen). Everhart Chaffee and Plante's results suggested that parents' ability stereotypes about language support girls' motivation for language arts; on the other hand, stereotypes that language arts are not for boys push them toward science. Boys are less interested in female-dominated fields, also regarding occupation, particularly when they feel pressure to conform to gender norms and hold stereotypical beliefs about these occupations (Masters and Barth). Endendijk and Portengen showed that parents' gender-typical career and family involvement (i.e., work hours and task division in the home) influence their children's vision of their future work and family roles. Children play an active role in developing this vision for the future through their gender identity, precisely by how similar they feel to individuals of the same gender.

Parental influence is so pervasive in children's acquisition of gender roles, knowledge, and understanding that perceived parenting styles are even related to young adults later intimate relationships outside the family. Paleari et al., in their study on cyber dating abuse, pointed out that the more young adults report that their mothers' parenting style was authoritarian or permissive during their childhood, the more likely they are to be involved in a cyber-abusive dating relationship. They have also found that mothers' parenting styles interact with fathers' styles in relating to their daughters' cyber control and aggression.

The studies included in this RT support the specific and interrelated role of fathers and mothers in children's gender socialization, substantially in the direction of conforming to gender stereotypes. In all these processes, children's sex and gender identity (Endendijk and Portegen) come into play by influencing parents' styles and practices and moderating their impact. Most gender disparities are harmful to girls at a young age, but some involve boys (see Everhart Chaffee and Plante), and they have long-term effects on academic paths, careers, and intimate relationships. It is nevertheless worthwhile noting that, under some individual and/or contextual conditions, the family can actively counteract cultural stereotypes about gender. For example, Shirefley and Leaper reported that highly educated parents—living near scientific/technology industries where women are employed—tend to use a higher proportion of science talk with daughters compared to sons.

These findings highlight that the psychosocial and educational programs to reduce the gender gap should start early at home by involving both parents. They could help parents to become more aware of their own gender-based biases and gender socialization practices, especially when these negatively impact children's health, by generating disparities (in terms of effective and symbolic opportunities), compromising children's (eudaimonic) wellbeing, and feeding feelings of unfairness across generations.

## Conclusion

Bringing together the above contributions, a multisystemic view of gender issues arises where different microsystems (mainly school and family) and sometime mesosystems (i.e., interactions across the microsystems) and macrosystems (i.e., cultures) are considered. This view can help in expanding focus to tap into a more comprehensive picture of gender differences and disparities and their consequences on youth's wellbeing in multiple daily life contexts so to inform social policies, provide intervention targets, and create a new community awareness of the roots of gender inequalities in current society.

Almost all the studies included in this RT provide a binary classification for gender. It would be worthwhile that future contributions on gender disparities in school and family contexts move beyond the binary toward a more multidimensional view of gender.

## Author contributions

CF and DB wrote the draft of this editorial. JE and JR contributed to revise it. All authors have read and approved the final manuscript.

## Conflict of interest

The authors declare that the research was conducted in the absence of any commercial or financial relationships that could be construed as a potential conflict of interest.

## Publisher's note

All claims expressed in this article are solely those of the authors and do not necessarily represent those of their affiliated organizations, or those of the publisher, the editors and the reviewers. Any product that may be evaluated in this article, or claim that may be made by its manufacturer, is not guaranteed or endorsed by the publisher.
